# Dyspnea induced by inspiratory loading limits dual-tasking in healthy young adults

**DOI:** 10.1371/journal.pone.0286265

**Published:** 2023-05-25

**Authors:** Stephanie R. Chauvin, Jessica Otoo-Appiah, Anna Zheng, Chris H. Ibrahim, James E. Ma, Dmitry Rozenberg, W. Darlene Reid

**Affiliations:** 1 Department of Physical Therapy, University of Toronto, Toronto, ON, Canada; 2 Respirology, Ajmera Transplant Center, University Health Network, Toronto, ON, Canada; 3 Temerty Faculty of Medicine, University of Toronto, Toronto, ON, Canada; 4 Interdepartmental Division of Critical Care Medicine, University of Toronto, Toronto, ON, Canada; 5 KITE—Toronto-Rehab-University Health Network, Toronto, ON, Canada; University of Ljubljana, SLOVENIA

## Abstract

**Objectives:**

Dyspnea is a common and multidimensional experience of healthy adults and those with respiratory disorders. Due to its neural processing, it may limit or interfere with cognition, which may be examined with a dual-task paradigm. The aim of this study was to compare single-task performance of Stroop Colour and Word Test (SCWT) or inspiratory threshold loading (ITL) to their combined dual-task performance. Secondly, whether mood was related to dyspnea or cognitive performance was also evaluated.

**Materials & methods:**

A virtual pre-post design examined single (SCWT and ITL) and dual-task (SCWT+ITL) performance. For ITL, a Threshold Trainer™ was used to elicit a “somewhat severe” rating of dyspnea. The SCWT required participants to indicate whether a colour-word was congruent or incongruent with its semantic meaning. The Depression, Anxiety and Stress Scale-21 (DASS-21) was completed to assess mood. Breathing frequency, Borg dyspnea rating, and breathing endurance time were ascertained.

**Results:**

Thirty young healthy adults (15F, 15M; median age = 24, IQR [23–26] years) completed the study. SCWT+ITL had lower SCWT accuracy compared to SCWT alone (98.6%, [97.1–100.0] vs 99.5%, [98.6–100.0]; p = 0.009). Endurance time was not different between ITL and SCWT+ITL (14.5 minutes, [6.9–15.0]) vs 13.7 minutes, [6.1–15.0]; p = 0.59). DASS-21 scores positively correlated with dyspnea scores during ITL (rho = 0.583, p<0.001) and SCWT+ITL (rho = 0.592, p<0.001).

**Conclusions:**

ITL significantly reduced dual-task performance in healthy young adults. Lower mood was associated with greater perceived dyspnea during single and dual-task ITL. Considering the prevalence of dyspnea in respiratory disorders, the findings of this dual task paradigm warrant further exploration to inform dyspnea management during daily activities.

## Introduction

Dyspnea is a common symptom of many pulmonary disorders such as chronic obstructive pulmonary disease (COPD) and interstitial lung disease (ILD) [1, 2]. It is highly prevalent with up to 27% of adults having experienced dyspnea [3]. In addition to dyspnea, patients with COPD have a prevalence of cognitive impairment ranging from ~10% to 60% [4, 5]. Common signs of impairment include changes in mood, difficulty completing multi-step tasks, and deficits in attention and executive functions (e.g. planning, decision making, and inhibition) [6, 7]. Cognitive impairments observed in chronic respiratory conditions have been attributed to smoking, hypoxia, depression, and poor cerebral blood flow [8].

Dyspnea is a multidimensional experience categorized by sensory and affective sensations like air hunger and distress [2, 9]. These affective sensations are further compounded by high rates of anxiety and depression in populations with chronic respiratory disease. Although the prevalence of anxiety and depression in COPD varies greatly in the literature, it has been reported to be as high as ~10% to 45% [10] and ~10% to 57% [11], respectively. Negative mood has also been correlated with increased perception of dyspnea severity [12, 13] which has implications for disease burden and treatment adherence [11, 14]. Consequently, dyspnea and mood can affect patients’ cognitive and physical function, especially when these factors interact.

Dual-tasking (i.e. ability to perform two tasks simultaneously), plays a major role in everyday life such as walking or driving while talking [15]. If tasks are cognitively demanding, competition for limited attentional resources leads to dual-task interference where performance in one or both tasks depreciates [15]. The effects of dual-task interference can be accentuated when cognitive capacity is impaired in disorders like COPD [16]. Due to the high attentional demands of dual-tasking, it is often used as a paradigm to evaluate cognitive interference with physical activities. Inspiratory muscle loading is a physical activity that not only requires motor control but may be further taxing cognitively because it induces the sensation of dyspnea.

Normally, respiration occurs automatically requiring no conscious attentional resources [17]. When respiratory demand increases and induces dyspnea, respiration becomes a conscious effortful process requiring cortical control [18, 19]. Overlapping need for cortical resources may lead to impairments in both cognitive performance and dyspnea tolerance. Although dyspnea has been suggested as an independent contributor to respiratory-associated cognitive impairment [13, 20, 21], evidence of cognitive interference from dyspnea is inconsistent. Inspiratory threshold loading (ITL) while completing a fear recognition task impaired facial recognition [22] but did not have an effect on accuracy of the Stroop Colour and Word Test (SCWT), which assesses executive function [23]. Moreover, induced dyspnea only led to deficits in SCWT accuracy of incongruent trials (i.e. colour-word and semantic word do not match) [24]. Heterogeneity among studies may be attributed to lower cognitive demand (30 trials [25], 80 trials [24] of the SCWT), shorter duration of dual-tasking (6.5 minutes [25], 5.3 minutes [24]), and unequal distribution of males and females (4F:8M [22], 26F:10M [24]). Addressing task difficulty, dual-tasking duration, and male/female ratio may aid in resolving the disparity observed in the literature.

The aim of this study was to compare single-task performance of SCWT and ITL to their combined dual-task performance. Secondly, whether mood was related to dyspnea or cognitive performance was examined. The current study is novel in its level of cognitive demand (208 trials of SCWT), its duration of dual-tasking (up to 15 minutes), and its equal ratio of males to females. Furthermore, this study was conducted virtually to increase accessibility for participants especially in the COVID environment and to alleviate stress associated with in-person research participation, which potentially influences baseline mood. We hypothesized that ITL demands would negatively affect dual-tasking performance in healthy young adults, and that mood and dyspnea would be positively correlated. Understanding these relationships may help inform management of patients with dyspnea or aid healthy adults to manage acute instances of breathlessness.

## Materials & methods

### Participants

This was a virtual pre-post design approved by the University of Toronto Health Sciences Research Ethics Board (00041701) and was conducted in accordance with the Declaration of Helsinki [26]. All participants provided signed informed consent, and demographic information and study results were deidentified. Thirty healthy young adults aged 18–35 (15 female, 15 male) were recruited through posters and social media posts at the University of Toronto. The sample size of 30 participants was calculated from an effect size of a previous report examining dyspnea and cognition [27], with an alpha of 0.05 and power of 0.80. Individuals were included in the study if they were fluent in English, had a computer with a webcam, and passed pre-screening using the American College of Sports Medicine exercise preparticipation screening questionnaire [28]. Participants were excluded from the study if they: were currently participating in competitive sports at the university, provincial and/or national level; had a diagnosis of an acute/chronic respiratory disorder; were colour blind; pregnant; had a history of unstable anxiety or panic attacks; or demonstrated signs and/or symptoms of cardiovascular, renal or metabolic diseases based on the American College of Sports Medicine screening questionnaire. Participants were also asked not to consume caffeinated and/or alcoholic beverages 4 hours and 24 hours, respectively, prior to study participation.

### Experimental protocol

This study examined single-tasks (SCWT and ITL) and a dual-task (simultaneous SCWT+ITL) in randomized order. The study was conducted in a single 1.5-hour session virtually utilizing Zoom Video Communication. Participants’ Modified Borg Dyspnea Scale (Borg) [18, 29] scores and breathing frequency (breaths/min) were collected at baseline prior to any tasks. After the familiarization phase to determine a “somewhat severe to very severe” Borg score, participants completed the Depression Anxiety and Stress Scale (DASS-21) [30].

Next, participants completed tasks in randomized order: 1) SCWT; 2) ITL using the Threshold Inspiratory Muscle Trainer™ (Philips Respironics, HS730EU-001), and 3) combined SCWT+ITL. Participants were provided a minimum two-minute rest between each task, or until they returned to their baseline Borg rating. The single-task SCWT was performed for 15 minutes. The single-task ITL and dual-task SCWT+ITL were performed for as long as participants could tolerate up to a maximum of 15 minutes. This maximum duration was chosen based on previous laboratory experience, and findings that 10 minutes of ITL decreased cognitive performance in healthy adults [24]. For the ITL and SCWT+ITL tasks, participants were encouraged to tolerate ITL for as long as possible but were instructed that they could stop at any point. Breathing frequency was determined by observing participants’ chest rise and fall, and validated by two raters during the last 15 seconds of each task [31]. Participants were asked to rate their level of breathlessness using the Borg scale [18, 29] and to select their top three descriptors of breathlessness using the Qualitative Dyspnea Assessment questionnaire [32, 33].

### Physical task by inspiratory threshold loading (ITL)

The physical task was induced using the Threshold Inspiratory Muscle Trainer™, a handheld device with an adjustable valve to target the threshold inspiratory load [34]. The Borg scale was used to quantify participants’ self-reported dyspnea [18, 29, 35]. The testing session began with a 10-minute familiarization phase where participants were oriented to the Threshold Inspiratory Trainer™. Participants were instructed to breathe through the device at the lowest load (9cmH_2_O) for two minutes. Inspiratory load was then incrementally increased every two minutes at a rate of 10–20% of participants’ predicted maximal inspiratory pressure (MIP) [36]. Participants completed the familiarization session if they: 1) reported a somewhat severe to severe (4-5/10) level of breathlessness [24]; or 2) reached the maximum inspiratory load of the device (41cmH_2_O). This inspiratory load was used to induce the respiratory demands in the subsequent tasks.

### The Stroop Colour and Word Test (SCWT)

A SCWT [37] using four-colour words was used as a cognitive task presented in PowerPoint on a computer monitor. Participants indicated whether the colour-word was congruent (e.g. the word “red” in red font) or incongruent (e.g. the word “red” in blue font) with the semantic meaning of the word by raising their hand and clicking an audible clicker. Twelve colour-word pairings were used for incongruent trials. Colour-words were presented in 52-point Arial font on a white background (median screen size 13 inches). Different versions of the SCWT were presented during the single and dual-tasks to avoid learners’ bias, and the order of presentation was counterbalanced across participants. Both the single and dual-task versions of the SCWT consisted of two blocks where participants were instructed to select congruent colour-word pairings and two blocks where participants were instructed to select incongruent colour-word pairings. Blocks were presented in an alternating format (A-B-A-B). Each block was pseudo-randomized between response and inhibition trials, and preceded by a 17-second instruction slide that provided explicit instructions to select congruent or incongruent colour-word pairings [38]. Participants were provided practice trials of the SCWT to ensure understanding. Fifty-two trials were presented in each block (3 min 30 sec) leading to a total of 208 trials in the single and dual-task phases of the study, respectively. Each trial was presented for two seconds followed by a two second fixation cross [24].

### Outcome measures

Dyspnea was assessed during each task using the Modified Borg Dyspnea Scale and Qualitative Dyspnea Assessment. The Borg scale [18, 29, 35] is a 0–10 numerical scale that has numbers corresponding to word descriptors of the intensity of dyspnea (e.g. nothing at all, moderate, severe, maximal) [35]. The Borg scale is a commonly used, reliable, and valid outcome measure to assess perceived dyspnea in a variety of populations [35, 39].

The Qualitative Dyspnea Assessment [32, 33] lists nineteen descriptors of breathlessness categorized into ten clusters. These qualitative dyspnea descriptors have been used to characterize exertional dyspnea during and following exercise in healthy young adults [32] and in individuals with ILD [40].

The DASS-21 is a 21-item self-report measure consisting of three subscales that measure depression, anxiety, and stress over a one-week timespan [30]. The DASS-21 is a short form of the DASS-42 and was created as a more manageable alternative to the full length version [41]. Each subscale consists of 7 items rated on a 4-point Likert scale with 0 corresponding to “did not apply to me at all” and 3 corresponding to “applied to me very much or most of the time”. Final scoring for each subscale is doubled to enable comparison to the original version. In addition to subscale scores, multiple studies have found strong support in using the total score for the DASS-21 for general emotional distress in both adolescents and young adults [42–45]. As such, summary scores across the three subscales were used in our analyses. The DASS-21 has shown to be valid and reliable in a healthy population ranging from 18 to 35 years [41, 42].

### Statistical analysis

Statistical analyses were conducted using IBM SPSS Statistics version 28. Descriptive statistics (medians and interquartile ranges (IQR)) were performed to summarize the sample. Wilcoxon signed-rank tests were used to compare the percent accuracy and number of trials completed of the SCWT to SCWT+ITL, and to compare the endurance time of ITL to SCWT+ITL. Mann Whitney U tests were used to identify differences between males and females in SCWT accuracy, SCWT+ITL accuracy, and SCWT+ITL trial completion. Friedman tests were used to examine: 1) an order effect for performance accuracy (%) and endurance time (minutes) of the randomized order of the SCWT, ITL, and SCWT+ITL; and 2) breathing frequency and Borg scores compared among baseline, SCWT, ITL, and SCWT+ITL tasks. If Friedman tests showed significance, post-hoc Wilcoxon signed-rank tests were used to further identify significant differences at an α value of ≤0.008, based on a Bonferroni correction. An α value of ≤0.05 was considered statistically significant for all comparisons except for the multiple post-hoc comparisons after the Friedman test.

Spearman correlations were used to determine the relationship between mood (summary score of the three DASS-21 subscales) and percent accuracy of the SCWT and SCWT+ITL, and the relationship between mood and Borg scores. Qualitative dyspnea descriptors were summarized by response frequency and a chi-square was used to examine for differences in descriptors among ITL familiarization, ITL single-task, and SCWT+ITL dual-task.

## Results

Thirty healthy young adults (15M, 15F) recruited for the study completed all tasks and had a median age of 24 [23–26] years and BMI of 23.4 [21.6–25.9] kg/m^2^ (**Table 1**). No adverse effects were reported.

**Table 1:** Participant demographics.

Characteristics (N = 30)	Median (IQR)	Min—max
**Age, y**	24 (23–26)	19–34
**Sex, n (male/female)**	15/15	-
**BMI, kg/m** ^**2**^	23.4 (21.6–25.9)	18.9–31.2
**DASS-21 score (Max score = 126)**	17 (7–26)	0–58
**Computer screen size, in**	13 (13–15)	11–32
**Predicted MIP, cmH**_**2**_ **O**	112.4 (84.5–127.5)	75.3–133.9
**MIP used, %** *	26 (20–32)	12–51

BMI = body mass index; DASS-21 = Depression, Anxiety, and Stress Scale-21; MIP = maximal inspiratory pressure

*MIP used is the threshold load required to induce a Borg score of 4.

MIP Equation [36] (Males): MIP = 126–1.029 × age + 0.343 × weight (kg) ± 22.4

MIP Equation [36] (Females): MIP = 171–0.694 × age + 0.861 × weight (kg)– 0.743 × height(cm) ± 18.5

**Fig 1:**
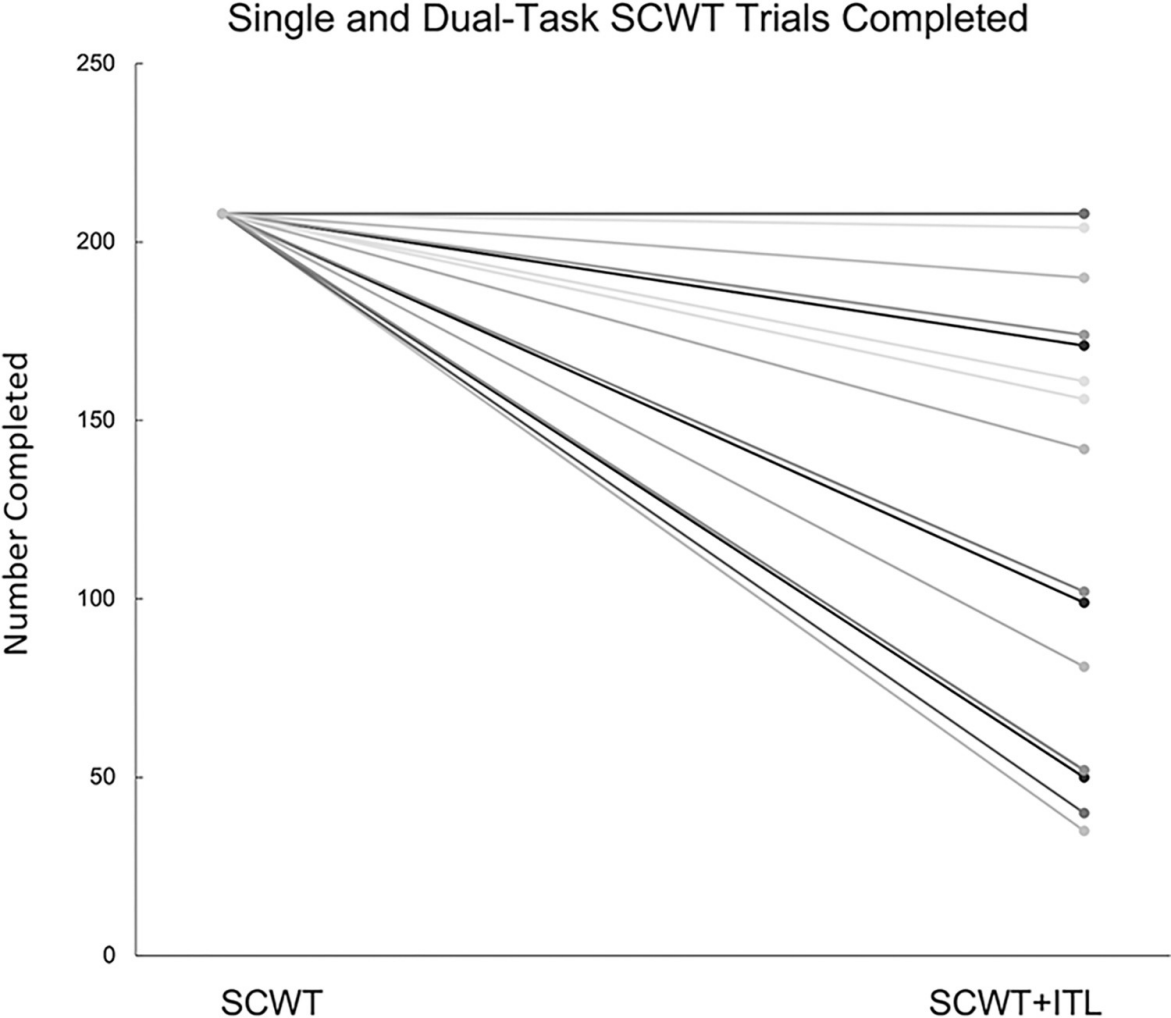
Distribution of the number of trials completed for the single-task Stroop Colour and Word Test (SCWT) [37] and the dual-task SCWT and Inspiratory Threshold Loading (SCWT+ITL). Each line represents one participant. Because some of the results are superimposed, 30 distinct lines are not apparent.

Dual-tasking SCWT+ITL resulted in significantly lower SCWT accuracy compared to the single-task SCWT (98.6 [97.1–100] vs 99.5 [98.6–100]%; p = 0.009; **Table 2**) and significantly fewer completed trials (197 [95–208] vs 208 [0]; p<0.001; **Fig 1**). There were no differences between males and females for single-task SCWT accuracy (M:100.0 [99.0–100.0] vs F:99.0 [98.0–100.0]%; p = 0.26), dual-task SCWT accuracy (M:100.0 [98.0–100.0] vs F:98.0 [97.0–99.0]%; p = 0.09), or dual-task SCWT trials completed (M:208.0 [102.0–208.0] vs F:161.0 [52.0–208.0]; p = 0.16). There were no differences between median accuracy for incongruent (colour-word did not match its semantic meaning) and congruent trials (colour-word matched its semantic meaning) in the single-task SCWT (congruent SCWT 100.0 [99.0–100.0]% vs incongruent SCWT 99.0 [98.1–100.0]%; p = 0.12), or the dual-task SCWT+ITL conditions (congruent SCWT+ITL 99.9 [98.0–100.0]% vs incongruent SCWT+ITL 98.1 [96.2–100.0]%; p = 0.11).

**Table 2:** Comparison of cognitive performance accuracy and time across single and dual-tasks.

	SCWT	ITL	SCWT+ITL	P-value
**Trials completed, #**	208 (208–208)	-	197 (95–208)	**<0.001** *
**Correct responses, %**	99.5 (98.6–100)	-	98.6 (97.1–100)	**0.009** *
**Time completed, min**	15.0 (15.0–15.0)	14.5 (6.9–15.0)	13.7 (6.1–15.0)	0.59

Presented as medians (IQR). SCWT = Stroop Colour and Word Test; ITL = inspiratory threshold loading.

46.7% and 50% of participants completed a maximum time of 15 minutes during ITL and SCWT+ITL, respectively.

*Significant at the p<0.05 level

Endurance time did not differ between single-task ITL and dual-task SCWT+ITL (14.5 [6.9–15.0] vs 13.7 [6.1–15.0] minutes, respectively; p = 0.59; **Table 2**).

Borg ratings were significantly higher during ITL and SCWT+ITL compared to baseline and SCWT levels (p<0.001; **Table 3**). However, Borg ratings did not differ between ITL and SCWT+ITL (4.8 [4.0–5.6] vs 5.0 [4.0–6.0], respectively; p = 0.32), respectively. Breathing frequency did not differ across baseline, SCWT, ITL, and SCWT+ITL (p = 0.699).

**Table 3:** Breathing frequency and Borg scores for baseline, single and dual-tasks.

	Baseline	SCWT	ITL	SCWT+ITL	P-value^a^
Breathing Frequency (breaths/min)	16.0 (12.0–18.5)	16.0 (13.5–16.0)	15.0 (8.0–20.0)	16.0 (12.0–18.0)	0.70
Borg Score	0.0	0.0	4.8 (4.0–5.6)*^†^	5.0 (4.0–6.0)*^†^	**<0.001**

Presented as medians (IQR). SCWT = Stroop Colour and Word Test; ITL = inspiratory threshold loading.

^a^ P-value from Friedman Test from comparisons across 4 conditions.

*Different from baseline at p≤0.008 from Wilcoxon Test.

^†^Different from SCWT at p≤0.008 from Wilcoxon Test.

**Fig 2:**
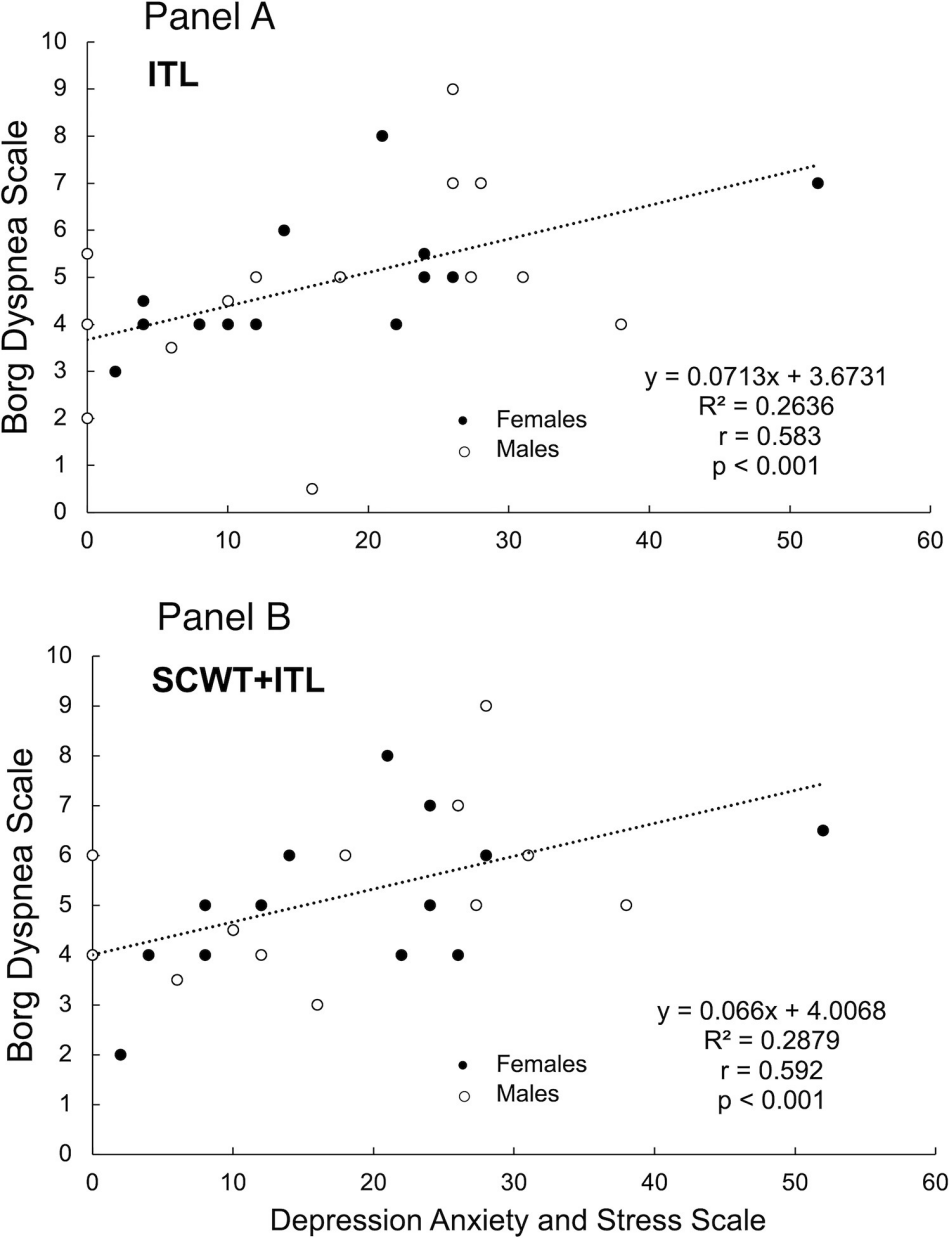
Relationship between the Depression Anxiety and Stress Scale (DASS-21) [30] and Borg [18, 29] scores during the single-task Inspiratory Threshold Loading (ITL) (Panel A) and dual-task Stroop Colour and Word Test [37] and ITL (SCWT+ITL) (Panel B). Outlier DASS-21 score of 58 removed to strengthen correlation as greater than 2 SD above the mean.

DASS-21 scores were correlated with Borg scores reported for ITL (rho = 0.504, p = 0.005) and SCWT+ITL (rho = 0.450, p = 0.01). Removal of a DASS-21 outlier score of 58 (greater than the median plus or minus three times the median absolute deviation [46, 47]), strengthened correlations for both ITL (rho = 0.583, p<0.001) and SCWT+ITL (rho = 0.592, p<0.001; **Fig 2**). DASS-21 scores were not correlated to SCWT accuracy during single-task SCWT or dual-task SCWT+ITL (p = 0.68; p = 0.74, respectively).

No order effect was found for SCWT accuracy (%) for single-task SCWT (p = 0.93) or dual-task SCWT+ITL (p = 0.86) performance, or endurance time for ITL (p = 0.44) or SCWT+ITL (p = 0.705).

**Fig 3:**
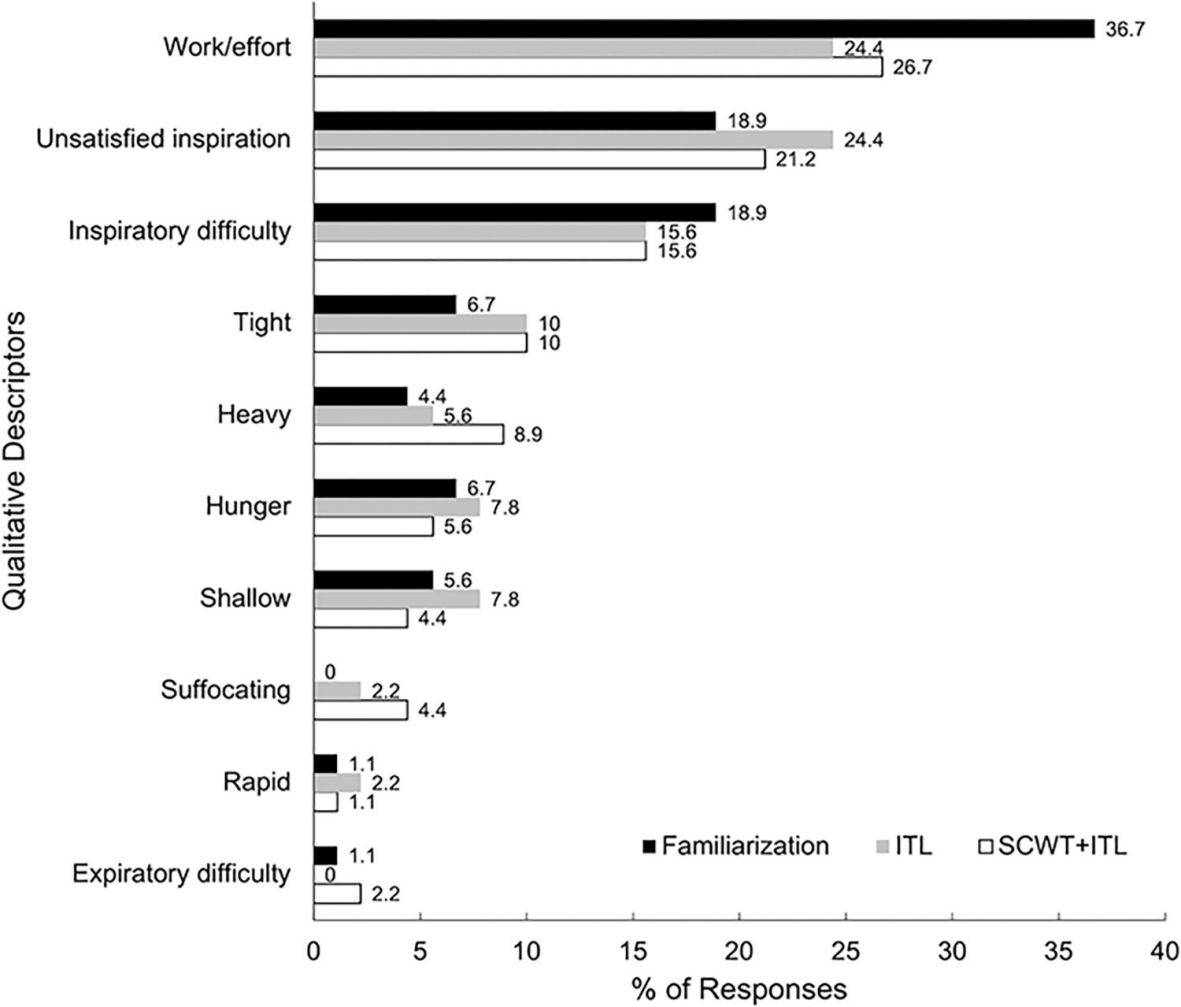
Frequency of qualitative dyspnea descriptors. These descriptors were reported during: the initial familiarization of inspiratory threshold loading (ITL), the single-task ITL, and the dual-task Stroop Colour and Word Test and ITL (SCWT+ITL). Each participant selected the top three descriptors that described their dyspnea. The percentage of responses is indicated to the right of each bar in addition to the horizontal axis.

During familiarization, ITL, and SCWT+ITL tasks, the top three descriptors most commonly reported, in order, were: 1) Work/effort, 2) Unsatisfied inspiration, and 3) Inspiratory difficulty (**Fig 3**). However, the frequency of the “work/effort” descriptor compared to all other descriptors did not differ among ITL familiarization, ITL single-task, and SCWT+ITL dual-task (p = 0.16).

## Discussion

The novel findings of this study indicate that the physical task of ITL negatively impacted dual-task performance in healthy young adults with no differential effect on male versus female participants. Improved cognitive accuracy and greater task completion were obtained for the single-task SCWT compared to the dual-task SCWT+ITL. Participants with higher DASS-21 scores reported higher levels of dyspnea during ITL and SCWT+ITL. Notably, participants consistently selected the same three qualitative dyspnea descriptors during ITL familiarization, single-task ITL, and dual-task SCWT+ITL tasks.

In the current study, we found decreased cognitive accuracy during dual-tasking (SCWT+ITL). Previous investigations showed that dual-tasking SCWT+ITL decreased accuracy for incongruent SCWT trials in one report [24] but had no effect on accuracy in another study of healthy participants [22, 24]. Our study may have consistently induced more demanding dual-tasking requirements because of a higher induction of inspiratory load and greater cognitive difficulty. Previous studies have used 160 trials (10.7 minutes) [24] or a 2-colour word version (2.7 minutes) [22] of the SCWT, which may not possess sufficient difficulty to induce dual-task interference. Our study consisted of a 208 trial 4-colour word SCWT, with dual-tasking lasting a maximum of 15 minutes which also exceeded the duration used in previous studies [24, 48, 49]. Furthermore, the ITL familiarization provided an adjustment orientation to ITL that enabled a targeted “somewhat severe to severe” load for participants whereas previous studies did not describe this feature.

The dual-task SCWT+ITL may have induced motor-cognitive interference due to the competing cortical demands of respiratory muscle motor control and affective neural processing of dyspnea. Dual-tasking ability may be tempered by the transition of breathing from an automatic process to a process mediated by cortical networks [50]. Increased mechanical loading of the respiratory muscles induces cortical activation [50] whereby more cognitive resources are delegated towards motor control of respiratory muscle recruitment to meet increased work of breathing demands. Consequently, this may decrease availability of cognitive resources for additional tasks and interfere with cognitive performance [20, 51]. During dual-tasking, cognitive resources may have been directed towards maintaining performance in the ITL task, leading to insufficient resources to manage the additional demands created by the SCWT. This may explain why ITL performance remained relatively stable while SCWT completion and accuracy decreased during dual-tasking [20, 52]. This effect is more pronounced when there is overlap in the neural processing required to complete both tasks as has been observed with ITL and the SCWT [53, 54]. Furthermore, increased dyspnea further compounds neural demands due to the need for its affective processing [50].

The dual-task limitations of SCWT+ITL may be explained by a capacity sharing model that provides a framework for central processing of dual-tasking. This model states that processing multiple tasks can be limited by cognitive resources, the allocation of which may be voluntary or be influenced by other characteristics such as respiratory mechanics, sex, and affect [55]. The success of dual-tasking performance depends on the ability to divide and direct attentional resources, as well as intact executive function [56]. As those with chronic respiratory disorders frequently experience cognitive impairment, they may have a reduced capacity to direct attention when cognition is challenged. This has been demonstrated by dual-task impairments of walking combined with spelling backwards in COPD patients [57, 58]. It would be of future relevance to examine dual-tasking of ITL combined with a cognitive task in this patient group.

Another finding of our study was the strong association between mood and dyspnea without experimental manipulation of mood, which aligns with previous research examining this association [59–61]. These relationships were shown during both single-task ITL and dual-task SCWT+ITL. Previously, low valence ratings (negative mood) experimentally induced by images (International Affective Picture System) was associated with higher ratings of dyspnea intensity and unpleasantness during a treadmill test in healthy participants [62]. Although the mechanism explaining this effect has not been elucidated, emerging neuroimaging supports an association between cortically controlled respiration and emotion. Respiratory driven brain oscillations have been observed during fear recognition and in brain areas associated with reward and motivation [63, 64]. Activation of the limbic system, which is involved in processing emotions, has also been demonstrated during ITL [65]. Individuals with chronic dyspnea experience higher rates of anxiety and depression which can lead to a more prominent perception of dyspnea than what is observed in a healthy population. The impact of mood on dyspnea may translate to failure of dyspnea management and increased disease burden for those who suffer from chronic dyspnea [66].

The three qualitative descriptors most frequently ranked were consistent among the ITL familiarization, single-task ITL, and dual-task SCWT+ITL: “work/effort”, “unsatisfied inspiration”, and “inspiratory difficulty”. After exercise testing in healthy adults, participants have described their breathing using the “work/effort” and “heavy” descriptors [40]. Furthermore, participants with lung diseases, such as COPD and ILD, consistently described their feelings of dyspnea following exercise using the terms “work/effort”, “inspiratory difficulty”, and “unsatisfied inspiration” [40, 67, 68], which were the same descriptors selected by participants undergoing dyspnea in the present study. This similarity of the top three descriptors in our study demonstrates that our methods using ITL were able to induce dyspnea that was of a similar qualitative nature to the dyspnea induced by exercise in patients with COPD and ILD.

An order effect was not found when comparing endurance times of ITL and SCWT+ITL, or performance accuracy of SCWT and SCWT+ITL. It can be hypothesized that the ITL familiarization session was successful in ensuring participants became acquainted with the ITL device without a learning or order effect. This is important for future research exploring dyspnea and dual-tasking to ensure a learning or order effect is mitigated by including a familiarization session.

## Limitations

This study was performed virtually over Zoom due to the COVID-19 environment. As a consequence of the virtual nature, there were limitations in assessing breathing frequency and inability to evaluate other ventilatory parameters such as respiratory muscle outcomes (electromyography and pressures) and minute ventilation during ITL. While the study was open to healthy young adults, recruitment primarily consisted of rehabilitation science students, a population which may have higher levels of exercise, stress, and cognition dissimilar to the general population [69]. Another limitation was the Threshold Inspiratory Trainer™ that has a maximum pressure of 41cmH_2_O, which did not elicit a somewhat severe to severe level of dyspnea in four participants. Future studies should consider evaluating reaction time to further quantify the impact of dyspnea on dual-tasking performance.

## Conclusion

Performing a cognitively demanding SCWT task in conjunction with ITL significantly reduced cognitive accuracy and performance in a healthy young adult population. Additionally, lower mood was associated with higher levels of perceived dyspnea. Given the prevalence of dyspnea and cognitive impairment in some respiratory disorders, future research evaluating the relationship among dyspnea, mood, and cognition in chronic lung disease patients may provide more informed rehabilitation strategies.

## Supporting information

S1 File Summary of descriptive statistics. (DOCX)
