# Supplementary material for: Dyspnea induced by inspiratory loading limits dual-tasking in healthy young adults
Source: PLoS One. 2023 May 25;18(5):e0286265. doi: 10.1371/journal.pone.0286265 (PMC10212117; doi:10.1371/journal.pone.0286265)
Supplement: S1 File — (DOCX) [file pone.0286265.s001.docx]

**SUMMARY OF DESCRIPTIVE STATISTICS**

Dyspnea induced by inspiratory loading limits dual-tasking in healthy young adults

*Stephanie R. Chauvin, Jessica Otoo-Appiah, Anna Zheng, Chris H. Ibrahim, James E. Ma, Dmitry Rozenberg, W. Darlene Reid*

**Demographics of Participants**

|  | Height (cm) | Weight (kg) | Sex | MIP | DASS-21 Score |
| --- | --- | --- | --- | --- | --- |
| Mean | 169.8 | 68.6 | 15M | 106.2 | 18.5 |
| Minimum | 154.0 | 52.0 | 15 F | 75.3 | 0.0 |
| Maximum | 188.0 | 90.0 |  | 133.9 | 58.0 |
| SD | 9.1 | 11.6 |  | 21.9 | 14.5 |
| Mode | 168.0 | 53.0 |  | 128.7 | 26.0 |
| Median | 168.5 | 69.0 |  | 112.4 | 17.0 |

DASS-21, Depression, Anxiety and Stress Scale-21; MIP, maximal inspiratory pressure

**Single and Dual Task Outcomes**

|  | Baseline | | Stroop Colour Word Test (SCWT) | | | | SCWT + ITL | | | | | ITL | | |
| --- | --- | --- | --- | --- | --- | --- | --- | --- | --- | --- | --- | --- | --- | --- |
|  | RR | Borg | RR | Borg | SCWTTrials | % Correct | RR | Borg | Trials | % Correct | Time (Min) | Time (Min) | RR | Borg |
| Mean | 15.5 | 0.1 | 15.3 | 0.1 | 208 | 99.1% | 15.6 | 5.1 | 155.8 | 97.6% | 11.3 | 10.8 | 15.3 | 4.9 |
| Minimum | 12.0 | 0.0 | 12.0 | 0.0 | 208 | 96.2% | 8.0 | 2.0 | 35.0 | 86.5% | 2.6 | 1.0 | 8.0 | 0.5 |
| Maximum | 22.0 | 0.5 | 20.0 | 1.0 | 208 | 100.0% | 30.0 | 9.0 | 208.0 | 100.0% | 15.0 | 15.0 | 32.0 | 9.0 |
| SD | 3.2 | 0.2 | 2.6 | 0.2 | 0 | 1.0% | 5.7 | 1.6 | 65.8 | 3.5% | 4.7 | 4.9 | 6.9 | 1.7 |
| Mode | 12.0 | 0.0 | 16.0 | 0.0 | 208 | 100.0% | 16.0 | 4.0 | 208.0 | 100.0% | 15.0 | 15.0 | 8.0 | 4.0 |
| Median | 16.0 | 0.0 | 16.0 | 0.0 | 208 | 99.5% | 16.0 | 5.0 | 197.0 | 98.6% | 14.5 | 13.7 | 15.0 | 4.8 |

ITL, inspiratory threshold loading
